# Association of Patient-Reported Outcomes and Nutrition with Body Composition in Women with Gynecologic Cancer Undergoing Post-Operative Pelvic Radiotherapy: An Observational Study

**DOI:** 10.3390/nu13082629

**Published:** 2021-07-29

**Authors:** Jie Lee, Tze-Chien Chen, Ya-Ting Jan, Chi-Jung Li, Yu-Jen Chen, Meng-Hao Wu

**Affiliations:** 1 Department of Radiation Oncology, MacKay Memorial Hospital, Taipei 104215, Taiwan; chijung1979@gmail.com (C.-J.L.); oncoman@mmh.org.tw (Y.-J.C.); radionc1@gmail.com (M.-H.W.); 2 Department of Medicine, MacKay Medical College, New Taipei City 252005, Taiwan; su27@ms11.hinet.net; 3 Department of Obstetrics and Gynecology, MacKay Memorial Hospital, Taipei 104215, Taiwan; 4 Department of Radiology, MacKay Memorial Hospital, Taipei 104215, Taiwan; gracilis0328@gmail.com; 5 Department of Biomedical Imaging and Radiological Sciences, National Yang-Ming University, Taipei 112304, Taiwan

**Keywords:** patient-reported outcome, body composition, pelvic radiotherapy, gynecologic cancer, nutrition

## Abstract

Pelvic radiotherapy is associated with gastrointestinal toxicities and deterioration of nutritional status. This study aimed to investigate the association of patient-reported outcomes (PROs) and nutritional status with body composition changes in women who underwent hysterectomy and post-operative radiotherapy for gynecologic cancer. We analyzed data of 210 patients treated with post-operative pelvic radiotherapy for gynecologic cancer between 2013 and 2018. The PRO version of the Common Terminology Criteria for Adverse Events (PRO-CTCAE) was used for gastrointestinal toxicity assessment. The Patient-Generated Subjective Global Assessment (PG-SGA) was used for nutritional assessment. Skeletal muscle index was measured from computed tomography scans at the L3 vertebral level. A reduction in skeletal muscle index ≥ 5% was classified as muscle loss. Odds ratios were calculated through logistic regression models. The PG-SGA score increased from the beginning to the end of radiotherapy (1.4 vs. 3.7, *p* < 0.001). Patients with PRO-CTCAE scores ≥ 3 had significantly higher PG-SGA scores at the end of radiotherapy than those with PRO-CTCAE scores ≤ 2 (8.1 vs. 2.3, *p* < 0.001). On multivariable analysis, PRO-CTCAE scores ≥ 3 and PG-SGA scores ≥ 4 at the end of radiotherapy were independently associated with increased risk of muscle loss (odds ratio: 8.81, *p* < 0.001; odds ratio: 72.96, *p* < 0.001, respectively). PROs and PG-SGA may be considered as markers of muscle loss after post-operative pelvic radiotherapy for gynecologic cancer.

## 1. Introduction

Post-operative pelvic radiotherapy is performed in women treated with hysterectomy for cervical or endometrial cancer with risk factors for recurrence [1,2,3,4,5]. Pelvic radiotherapy is associated with gastrointestinal (GI) toxicities, including diarrhea, abdominal pain, food intolerance, and fecal incontinence [6]. GI toxicities are burdensome to patients, interfere with the quality of life, and can lead to nutritional status deterioration and muscle loss [7,8,9,10,11,12]. Muscle loss can, in turn, affect outcome and quality of life in women with gynecologic cancer [10,11,12,13,14,15]. Accurate assessment of GI toxicities and nutritional status may help predict muscle loss and enable potential intervention to maintain muscle mass during pelvic radiotherapy [16].

Patient-reported outcomes (PROs) allow patients to self-report important clinical information, such as their symptoms (e.g., abdominal pain, diarrhea), and provide more accurate information of treatment-related toxicity. In clinical practice, clinicians commonly evaluate and quantify treatment-related toxicities using the Common Terminology Criteria for Adverse Events (CTCAE). However, disagreement between clinician-reported and patient-reported symptomatic toxic effects had been described [17]. To improve the reliability of capturing treatment-related toxicities, the National Cancer Institute has developed the PROs version of the CTCAE (PRO-CTCAE) that complements the CTCAE. The PRO-CTCAE is a survey based on a subset of the CTCAE items to characterize the frequency and severity of treatment toxicities and the extent to which these toxicities interfere with daily activities from the patient′s perspective [18]. Previous studies revealed that PRO-CTCAE could provide more accurate treatment-related toxicity assessments than CTCAE estimates from clinicians [8,19,20,21,22,23]. Hence, patient-reported toxicity may help increase the window of opportunity for clinicians to intervene and enhance supportive care to prevent muscle loss. However, data regarding relationships between patient-reported toxicity and body composition changes during pelvic radiotherapy are lacking.

The scored Patient-Generated Subjective Global Assessment (PG-SGA) is an adaptation of the SGA, validated for nutritional assessment in cancer patients [24]. The PG-SGA is composed of two parts: the first is a patient-reported assessment of the patient′s weight history, food intake, nutritional impact symptoms, and function; the second assesses disease, metabolic stress, muscle status, fat deposits, and fluid status and is conducted by a trained researcher, resulting in a score. A previous study reported that the nutritional status of cervical cancer patients deteriorates during pelvic radiotherapy using PG-SGA [10]. Hence, evaluating nutritional status by PG-SGA may play a role in predicting muscle loss after pelvic radiotherapy for these patients.

**Figure 1:**
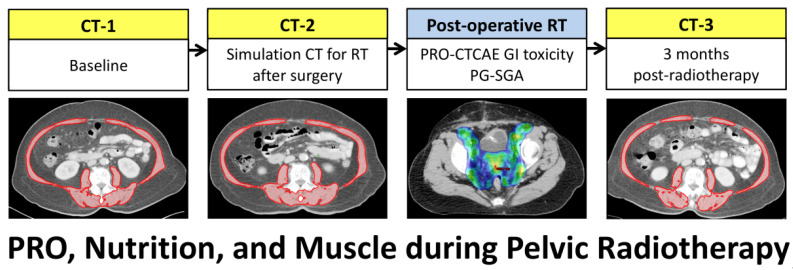
Scheme of CT scans for patients with cervical or endometrial cancer receiving surgery and post-operative pelvic radiotherapy. Skeletal muscle area (red) was measured on a CT slice at the L3 vertebral level. RT, radiotherapy.

Body composition changes can be longitudinally evaluated on computed tomography (CT) scans acquired for routine cancer care (Figure 1) [25,26,27,28,29]. This study aimed to evaluate whether patient-reported GI toxicity and nutritional status are associated with body composition changes in women who underwent pelvic radiotherapy following hysterectomy for cervical or endometrial cancer.

## 2. Materials and Methods

### 2.1. Patients

This retrospective study was conducted with the approval of the institutional review board. Informed consent was waived because of the retrospective nature of the study. We reviewed the data of women with cervical or endometrial cancer receiving post-operative radiotherapy after hysterectomy at our institution between 2013 and 2018. The inclusion criteria were: (a) sufficient clinical data, PRO-CTCAE data, and PG-SGA data; (b) CT before hysterectomy; (c) simulation CT for post-operative radiotherapy planning;, and (d) CT within 3 months after radiotherapy. Patients with history of malignancy were excluded.

### 2.2. Treatments

Pre-treatment CT was routinely performed before surgery (Figure 1). The surgery included hysterectomy, bilateral salpingo-oophorectomy, and lymphadenectomy. All surgeries were performed by accredited gynecologic oncologists. For patients with risk factors for recurrence, post-operative pelvic radiotherapy was indicated. Concurrent cisplatin-based chemotherapy was considered based on major risk factors (e.g., positive pelvic lymph node or parametrial invasion). A simulation CT was acquired for radiotherapy planning after favorable healing of the surgical wound. Pelvic radiotherapy was delivered using intensity-modulated radiotherapy technique. The total prescription dose was 45 or 50.4 Gy to the pelvic nodal region and upper vagina. High-dose-rate vaginal cuff brachytherapy, consisting of 5 Gy for 4–6 fractions, was administered at the discretion of the treating physicians. Post-radiotherapy CT was performed within 3 months after completion of radiotherapy.

### 2.3. Toxicity Assessment

Treating physicians evaluated toxicity weekly using the CTCAE version 4.0 during radiotherapy. Patient-reported toxicities were scored by patients using the PRO-CTCAE. Patients were provided PRO-CTCAE questionnaires regarding GI toxicities to assess the severity of abdominal pain, interference of abdominal pain with daily activities, and frequency of diarrhea for the record. Patients could record their symptoms in the questionnaires at home or whenever severe symptoms occurred. Patients then provided these PRO-CTCAE questionnaires before their weekly clinic appointments. The PRO-CTCAE items of diarrhea and abdominal pain were selected for the survey because these symptoms are the most common and clinically important GI toxicities experienced by patients during pelvic radiotherapy [30]. PRO-CTCAE was scored based on a 5-point Likert scale concerning severity (none to very severe), interference (not at all to very much), and frequency (never to almost constantly), with 0 indicating none, not at all, and never, respectively.

### 2.4. Nutritional Assessment

Patients’ nutritional status was assessed using PG-SGA at the beginning and end of radiotherapy in our clinical practice by trained personnel. On completion of the assessment, patients were subjectively categorized as A (well-nourished), B (suspected malnutrition or moderately malnourished), or C (severely malnourished). For this analysis, patients were divided into two groups according to the PG-SGA score—scores 0–3 and scores ≥4, with the latter group considered to be at risk of malnutrition [31,32].

### 2.5. Body Composition on CT Scans

Body composition was measured on CT scans at the L3 level. Body composition was defined based on Hounsfield unit (HU) thresholds, which ranged from −29 to + 150 HU for skeletal muscle, −50 to −150 HU for visceral adipose tissue, and −30 to −190 HU for subcutaneous adipose tissue. Cross-sectional areas (cm^2^) of the skeletal muscle and visceral and subcutaneous adipose tissues were measured on a single slice of CT by using the Varian Eclipse software (Varian Medical Systems Inc., Palo Alto, CA, USA) [25,26,27,28]. The sum of the areas of the visceral and subcutaneous adipose tissues was calculated to be total adipose tissue. The body composition was measured by one researcher blinded to clinical information and outcomes of patients. The cross-sectional areas were normalized for the patient height and reported as skeletal muscle index (SMI, cm^2^/m^2^) and total adipose tissue index (TATI, cm^2^/m^2^) [11]. Body mass index (BMI) within 2 weeks of the CT scans were obtained from medical records.

According to the current definition of cachexia: patients with weight loss >5% over the past 6 months [33] and with a decrease of ≥5% in BMI, SMI, or TATI after surgery and post-operative radiotherapy were considered to have weight loss, muscle loss, or adipose tissue loss, respectively. Previous studies also have reported that weight or muscle loss ≥ 5% is associated with a poor outcome in cancer patients [34,35,36,37].

We also analyzed systemic inflammation and bowel radiation dose-volume because these factors may affect body composition. We obtained data regarding the neutrophil-lymphocyte ratio (NLR) as a systemic inflammatory marker from medical records. NLR was categorized as high or low using a cut-off of 3 [38,39]. The bowel V45, which indicates the bowel volume (mL) receiving a radiation dose of ≥45 Gy [9], was obtained from the patient’s radiotherapy plan.

### 2.6. Statistical Analysis

The patient characteristics and clinical factors were presented as median and interquartile range (IQR) or mean ± standard deviation for continuous data and as numbers and percentages for categorical data. Continuous variables were compared using independent *t*-tests or Mann–Whitney U tests. The changes in body composition indexes across three timepoints were analyzed using repeated measures analysis of variance (ANOVA) with Bonferroni adjustment for post hoc tests. Spearman correlation coefficient was used to evaluate correlations.

The highest score for each question during 3–5 weeks of radiotherapy was used to analyze the PRO-CTCAE, and for the CTCAE, the highest grade was used. The toxicities during 3–5 weeks were analyzed because radiotherapy-related GI toxicities gradually increase to become symptomatic at 3 weeks and reach a maximum at 5 weeks [40]. McNemar’s test was used to evaluate the difference in physician-reported toxicity (CTCAE grade ≥ 3) and patient-reported toxicity (PRO-CTCAE score of 3–4).

Logistic regression models were used to evaluate the association of clinical factors with weight, muscle, and adipose tissue loss after radiotherapy. The results were presented as odds ratios (OR) with 95% confidence intervals (CI). All variables with *p* < 0.05 in univariable analysis were included in the multivariable analysis. The data were analyzed using IBM SPSS software (version 21.0; IBM Corp., Armonk, NY, USA). A *p*-value < 0.05 was considered to indicate statistical significance.

## 3. Results

### 3.1. Patient Characteristics

A total of 303 patients with cervical or endometrial cancer with indications for postoperative radiotherapy after hysterectomy were reviewed. The following patients were then excluded from analysis: patients with a history of malignancy (*n* = 6), missing required clinical data (*n* = 10), missing PRO-CTCAE data (*n* = 50), missing PG-SGA data (*n* = 16), CT not performed within three months after radiotherapy (*n* = 8), and CT scans of insufficient quality (*n* = 3). Finally, 210 patients were enrolled for analysis.

The patient and tumor characteristics are summarized in Table 1. All patients completed the planned pelvic radiotherapy with a median duration of 37 days (IQR: 35–40). The median follow-up period was 3.5 years (IQR: 2.5–5.6).

### 3.2. PRO-CTCAE and Physician-Reported CTCAE

Overall, 48 (22.9%) patients reported a PRO-CTCAE score ≥3 for GI toxicities, while 16 (7.6%) patients were reported by physicians to have physician-reported CTCAE grade ≥3 (*p* < 0.001). The physician-reported grade ≥3 abdominal pain rate was 3.8%, whereas 9.5% of women reported severe or very severe abdominal pain, and 9.0% reported that their abdominal pain interfered with their activities quite a bit or very much (*p* = 0.02 and 0.03, respectively). The physician-reported grade ≥3 diarrhea rate was 5.7%, whereas 20.5% of patients reported frequent or almost constant diarrhea (*p* < 0.001). Physician-reported grade ≥3 abdominal pain or diarrhea correlated with the corresponding PRO-CTCAE items (severity: ρ = 0.19, *p* = 0.006; interference: ρ = 0.19, *p* = 0.004; diarrhea: ρ = 0.20, *p* = 0.004).

Comparing patients with chemotherapy (*n* = 84) and without chemotherapy (*n* = 126), 22 (26.2%) and 26 (20.2%) patients reported PRO-CTCAE score ≥3 for abdominal pain or diarrhea (*p* = 0.35), respectively. In terms of CTCAE, 9 (10.7%) and 7 (5.6%) patients had grade ≥3 abdominal pain or diarrhea, respectively (*p* = 0.17).

### 3.3. PG-SGA Score at the Beginning and End of Radiotherapy

The PG-SGA score increased from the beginning to the end of radiotherapy (1.4 vs. 3.7, *p* < 0.001). The number of patients with PG-SGA score ≥4 was 29 (13.8%) at the beginning of radiotherapy and increased to 76 (36.2%) at the end of radiotherapy (*p* < 0.001). Patients with PRO-CTCAE score ≥3 had significantly higher PG-SGA scores at the end of radiotherapy than those with PRO-CTCAE score ≤2 (8.1 vs. 2.3, *p* < 0.001). However, the PG-SGA score at the end of radiotherapy was not significantly different between patients with physician-reported CTCAE grade ≥3 and grade ≤2 (5.1 vs. 3.5, *p* = 0.08).

Comparing patients with chemotherapy (*n* = 84) and without chemotherapy (*n* = 126), the change of PG-SGA score from the beginning to the end of radiotherapy was not significant between them (2.6 vs. 2.1, *p* = 0.56). PG-SGA scores at the end of radiotherapy were also not significantly different between them (3.8 vs. 3.5, *p* = 0.56).

### 3.4. Body Composition Changes after Surgery and Post-Operative Radiotherapy

**Figure 2:**
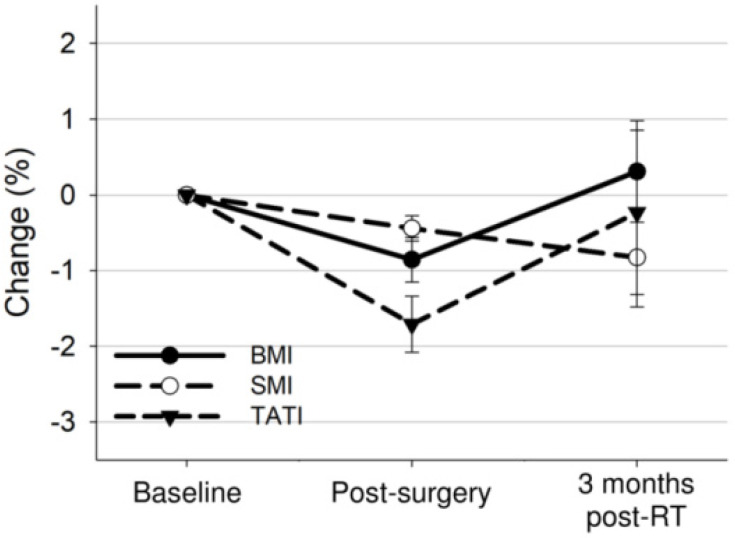
Overall mean changes with 95% confidence interval bars in BMI, SMI, and TATI from baseline to 3 months after treatment completion.

The median time from CT at baseline to simulation CT for radiotherapy and CT within three months post radiotherapy were 22 days (IQR: 20–24) and 136 days (IQR: 122–148), respectively. Our analysis revealed changes in BMI, SMI, and TATI across the three timepoints (*p* = 0.001, *p* = 0.03, *p* = 0.003, respectively) (Figure 2). BMI decreased from the baseline level by 0.9% after surgery (25.3 kg/m^2^ vs. 25.1 kg/m^2^, a reduction of 0.2 kg/m^2^; 95% CI: −0.3 to −0.2; *p* < 0.001) and returned to the baseline level three months post-radiotherapy. SMI decreased from the baseline level by 0.4% after surgery (40.0 cm^2^/m^2^ vs. 39.8 cm^2^/m^2^, a reduction of 0.2 cm^2^/m^2^; 95% CI: −0.2 to −0.1; *p* < 0.001) and by 0.8% three months post radiotherapy (40.0 cm^2^/m^2^ vs. 39.6 cm^2^/m^2^, a reduction of 0.3 cm^2^/m^2^; 95% CI: −0.6 to −0.1; *p* = 0.01). TATI decreased from the baseline level by 1.7% after surgery (115.5 cm^2^/m^2^ vs. 113.2 cm^2^/m^2^, a reduction of 2.3 cm^2^/m^2^; 95% CI: −2.8 to −1.9; *p* < 0.001) and by 0.2% 3 months post radiotherapy (115.5 cm^2^/m^2^ vs. 114.2 cm^2^/m^2^, a reduction of 1.3 cm^2^/m^2^; 95% CI: −2.6 to 0.1; *p* = 0.06). Changes in BMI were correlated to changes in SMI and TATI (SMI: ρ = 0.54; *p* < 0.001; TATI: ρ = 0.88; *p* < 0.001). After surgery and post-operative pelvic radiotherapy, 32 (15.2%), 40 (19.0%), and 66 (31.4%) patients developed ≥5% loss of weight, muscle, or adipose tissue, respectively.

Comparing patients with chemotherapy (*n* = 84) and without chemotherapy (*n* = 126), the change of body composition indexes was not significantly different between them (BMI: 0.4% vs. 0.3%, *p* = 0.92; SMI: −1.4% vs. −0.4%, *p* = 0.15; TATI: 0.1% vs. −0.4%, *p* = 0.72).

### 3.5. Body Composition Changes by PRO-CTCAE or Physician-Reported CTCAE

The body composition changes after surgery and post-operative radiotherapy according to PRO-CTCAE or physician-reported CTCAE are presented in Table 2. There were more patients with weight loss, muscle loss, or adipose tissue loss in the PRO-CTCAE score ≥3 group. However, the number of patients with weight, muscle, or adipose tissue loss was not different based on physician-reported CTCAE.

**Figure 3:**
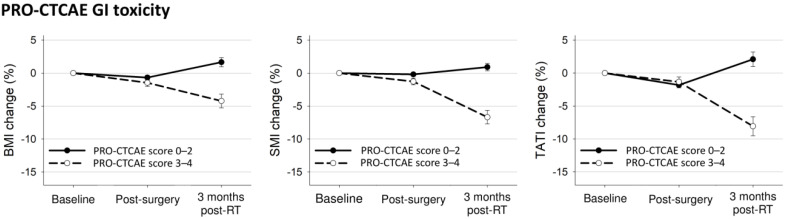
Mean changes with 95% confidence interval bars in body composition indexes from baseline to 3 months after treatment completion according to any PRO-CTCAE GI toxicity.

Figure 3 shows the body composition changes according to the PRO-CTCAE. Patients with PRO-CTCAE scores ≥3 had significantly decreased BMI and SMI after surgery (BMI: −1.5% vs. −0.7%, *p* = 0.03; SMI: −1.3% vs. −0.2%, *p* < 0.001; TATI: −1.3% vs. −1.9%, *p* = 0.27) and these body composition indexes after radiotherapy (BMI: −4.2% vs. 1.7%, *p* < 0.001; SMI: −6.7% vs. 0.9%, *p* < 0.001; TATI: −8.1% vs. 2.1%, *p* < 0.001) compared to patients with PRO-CTCAE scores ≤2 (Figure 3). On categorizing the patient population based on the diarrhea frequency or abdominal pain, patients with PRO-CTCAE scores ≥3 for these items showed a significant decrease in body composition indexes after radiotherapy compared to those in patients with PRO-CTCAE scores ≤2 (Supplementary Figure S1).

### 3.6. Body Composition Changes by PG-SGA

The body composition changes according to PG-SGA are presented in Table 3. The PG-SGA at the beginning of radiotherapy was not associated with weight, muscle, or adipose tissue loss. However, the PG-SGA at the end of radiotherapy was associated with weight loss, muscle loss, or adipose tissue loss after radiotherapy.

**Figure 4:**
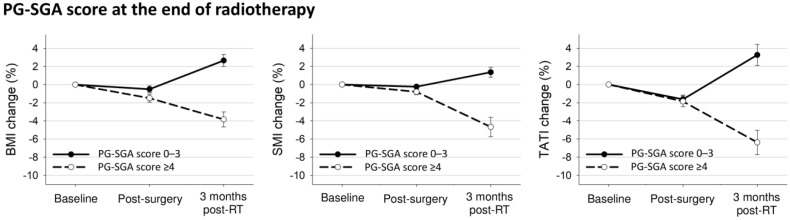
Mean changes with 95% confidence interval bars in body composition indexes from baseline to 3 months after treatment completion according to PG-SGA at the end of radiotherapy.

Figure 4 shows the body composition changes according to the PG-SGA at the end of radiotherapy. Patients with PG-SGA score ≥4 had decreased BMI and SMI after surgery (BMI: −1.5% vs. −0.5%, *p* = 0.001; SMI: −0.8% vs. −0.3%, *p* = 0.003; TATI: −1.9% vs. −1.6%, *p* = 0.56) and further decreased BMI, SMI, and TATI after radiotherapy (BMI: −3.8% vs. 2.7%, *p* < 0.001; SMI: −4.7% vs. 1.4%, *p* < 0.001; TATI: −6.4% vs. 3.3%, *p* < 0.001) compared to patients with PG-SGA score ≤3.

### 3.7. Predictor of Weight, Muscle, or Adipose Tissue Loss

Univariable logistic regression analysis revealed that NLR, PG-SGA score ≥4 at the end of radiotherapy, PRO-CTCAE items (any, diarrhea frequency, and abdominal pain interference), and bowel V45 were associated with weight, muscle, or adipose tissue loss (Supplementary Table S1). PRO-CTCAE abdominal pain severity was associated with muscle or adipose tissue loss but not weight loss. Age, disease site, surgical type, use of chemotherapy, physician-reported CTCAE, and PG-SGA at the beginning of radiotherapy were not associated with weight, muscle, or adipose tissue loss.

On multivariable logistic regression analysis adjusting for NLR and bowel V45, PG-SGA score ≥4 at the end of radiotherapy, PRO-CTCAE score ≥3 for diarrhea frequency, and abdominal pain interference were independently associated with increased risks of weight, muscle, or adipose tissue loss (Table 4). PRO-CTCAE abdominal pain severity was independently associated with an increased risk of muscle loss; however, it was not associated with weight or adipose tissue loss.

## 4. Discussion

This is the first study to evaluate the association of patient-reported GI toxicity and nutritional status with body composition changes in women who underwent radiotherapy following hysterectomy for cervical or endometrial cancer. We found that the nutritional status of patients deteriorated at the end of post-operative radiotherapy, and the deterioration in nutritional status was associated with patient-reported GI toxicity. We also found that PRO-CTCAE items scores ≥3 or PG-SGA scores ≥4 at the end of radiotherapy were associated with weight, muscle, or adipose tissue loss after radiotherapy. Physician-reported GI toxicity and PG-SGA score at the beginning of radiotherapy were not predictive of body composition loss after radiotherapy.

Pelvic radiotherapy is associated with GI toxicity and deterioration of the nutritional status of patients [10,11,12]. We found that the PG-SGA score at the end of radiotherapy was associated with the PRO-CTCAE score but not physician-reported CTCAE. The possible explanation is that both PG-SGA and PRO-CTCAE evaluated symptoms from the patient′s perspective. PG-SGA incorporates patient-reported weight, symptoms, food intake, and activity as parts of the assessment of patients’ nutritional statuses. The PRO-CTCAE characterizes the frequency and severity of treatment-related toxicities and the extent to which these toxicities interfere with a patient’s daily life. Moreover, previous studies have shown that patient-reported outcomes may provide a more accurate assessment of symptomatic treatment-related toxicity [19,20,21,22]. There is also evidence to suggest that, compared with clinician reporting, patient-reported outcomes are more strongly correlated with clinical outcomes [41,42]. These findings suggested the relevance of using patient-reported outcomes during cancer care to evaluate nutritional status and treatment-related toxicity more precisely.

Body composition changes in cancer patients are dynamic during treatment. A longitudinal evaluation of nutritional assessment can be important in cancer care [16]. In this study, all patients underwent post-operative pelvic radiotherapy for gynecologic cancer. We found that the PG-SGA score increased from the beginning to the end of post-operative radiotherapy. The number of patients with a PG-SGA score ≥4 also increased. Notably, the PG-SGA at the beginning of radiotherapy was not associated with body composition changes after radiotherapy, while that at the end of radiotherapy was independently associated with weight, muscle, or adipose tissue loss after radiotherapy. To prevent deterioration of nutritional status during pelvic radiotherapy, multimodal interventions, including nutrition, exercise, or anabolic medications, may help [16,43,44]. The length of nutritional intervention can also affect nutritional status and muscle because it can take months to restore muscle loss [43]. Pharmacologic mitigators to decrease pelvic radiotherapy-related toxicity may also help prevent malnutrition [9,45]. Future studies are needed to evaluate the effects of multimodal interventions in patients undergoing pelvic radiotherapy.

The optimal cut-off values of muscle loss with clinical significance are unclear. In this study, we used the 5% change to simulate the definition of cachexia [33]. Previous studies also reported that muscle loss ≥5% during treatments was associated with poorer survival outcomes in cancer patients [34,35,36]. However, the use of 5% should be further validated. One previous, large population study evaluating the prognostic impact of muscle loss during treatment in colorectal cancer suggested the analytic technique using standard deviation thresholds can be applied to identify patients with significant muscle wasting [46]. In their study, the average change in muscle mass was 0.1% ± 5.7% after treatment, and a cut-off value of 5.7% was slightly higher than the 5%. Although the 5% may not be an optimal cut-off value for muscle loss, it simulates the current definition of cachexia and may be reasonable in this study.

Chemotherapy may affect GI toxicity and nutritional status. The Gynecologic Oncology Group−109 trial compared concurrent cisplatin-based chemoradiotherapy and pelvic radiotherapy alone in patients treated with hysterectomy for cervical with high-risk factors for recurrence [4]. They reported that there were more patients with GI toxicities in the chemoradiotherapy arm. However, we found that the PRO-CTCAE, PG-SGA, and body composition changes were not different between patients with chemotherapy and without chemotherapy. The possible explanation might be that patients in this study received intensity-modulated radiotherapy that can deliver focused radiation to the pelvic lymphatic regions while minimizing doses to the bowel and therefore lowering the GI toxicity [6,7,8]. However, it should be noted that the GI toxicity of pelvic radiotherapy is mainly abdominal pain and diarrhea, while nausea and vomiting are mainly related to chemotherapy [30]. Despite using intensity-modulated radiotherapy, patients with chemotherapy may have more nausea and vomiting. The association of nausea and vomit with body composition needs to be evaluated in future studies.

This study had some limitations. First, it is a retrospective investigation with a small number of patients. Second, the study does not have the statistical power to evaluate the optimal timing of the PRO-CTCAE score to predict muscle loss. Third, the PG-SGA before surgery was not available for analysis due to the study′s retrospective design. Furthermore, quality-of-life evaluation using widely validated questionnaires was also lacking; hence, the effect of the quality of life on body composition changes is unknown. Selection bias and residual and unmeasured confounding are also potential limitations of this retrospective study. Our findings need to be validated in studies with larger cohorts. Despite these limitations, these patients received current standard treatments with adequate follow-up, and their outcomes are comparable to previous studies [6,7,8].

## 5. Conclusions

This study demonstrated that patient-reported GI toxicity and nutritional status at the end of radiotherapy are associated with weight, muscle, or adipose tissue loss after post-operative pelvic radiotherapy in women with gynecologic cancer. Physician-reported GI toxicity was not associated with body composition changes. Integrating the PRO-CTCAE or PG-SGA into clinical practice can help identify patients who may develop muscle loss after radiotherapy. Future studies are needed to investigate whether the PRO-CTCAE or PG-SGA guided multimodal intervention can preserve skeletal muscles in these patients.

## Figures and Tables

**Table 1:** Patient and tumor characteristics.

Characteristics	Overall (*n* = 210)
Age (years)	56 (50–62)
Disease cite	
Endometrium	142 (67.6)
Cervix	68 (32.4)
Surgery type	
Open	164 (78.1)
Minimally invasive	46 (21.9)
Radiation dose	
45 Gy	95 (45.2)
50.4 Gy	115 (54.8)
Brachytherapy	
Yes	142 (67.6)
No	68 (32.4)
Chemotherapy	
Yes	84 (40.0)
No	126 (60.0)
NLR	
≤3	134 (63.8)
>3	76 (36.2)
Bowel radiation dose-volume ^a^ V45 (mL)	158.9 (122.9–190.3)

Data are median (IQR) or *n* (%). ^a^ V45 = volume (mL) of bowel receiving 45 Gy or more.

**Table 2:** Body composition change groups after treatment by PRO-CTCAE or physician-reported CTCAE.

Variable	PRO-CTCAE Score			Physician-Reported CTCAE Grade		
	0–2 (*n* = 162)	3–4 (*n* = 48)	*p*-Value	0–2 (*n* = 194)	3–4 (*n* = 16)	*p*-Value
BMI change, *n* (%)						
Gain or loss <5%	148 (91.4)	30 (62.5)	<0.001	166 (85.6)	12 (75.0)	0.28
Loss ≥5%	14 (8.6)	18 (37.5)		28 (14.4)	4 (25.0)	
SMI change, *n* (%)						
Gain or loss <5%	150 (92.6)	20 (41.7)	<0.001	158 (81.4)	12 (75.0)	0.51
Loss ≥5%	12 (7.4)	28 (58.3)		36 (18.6)	4 (25.0)	
TATI change, *n* (%)						
Gain or loss <5%	130 (80.2)	14 (29.2)	<0.001	133 (68.6)	11 (68.8)	0.99
Loss ≥5%	32 (19.8)	34 (70.8)		61 (31.4)	5 (31.3)	

**Table 3:** Body composition change groups after treatment by PG-SGA.

Variable	PG-SGA at the Beginning of Radiotherapy			PG-SGA at the End of Radiotherapy		
	≤3 (*n* = 181)	≥4 (*n* = 29)	*p*-Value	≤3 (*n* = 134)	≥4 (*n* = 76)	*p*-Value
BMI change, *n* (%)						
Gain or loss <5%	153 (84.5)	25 (86.4)	1.00	133 (99.3)	45 (59.2)	<0.001
Loss ≥5%	28 (15.5)	4 (13.8)		1 (0.7)	31 (40.8)	
SMI change, *n* (%)						
Gain or loss <5%	149 (82.3)	21 (72.4)	0.21	133 (99.3)	37 (48.7)	<0.001
Loss ≥5%	32 (17.7)	8 (27.6)		1 (0.7)	39 (51.3)	
TATI change, *n* (%)						
Gain or loss <5%	125 (69.1)	19 (65.5)	0.67	116 (86.6)	28 (36.8)	<0.001
Loss ≥5%	56 (30.9)	10 (34.5)		18 (13.4)	48 (63.2)	

**Table 4:** Multivariable logistic regression analysis of factors associated with body composition changes.

Variable	Weight Loss ≥5%		Muscle Loss ≥5%		Adipose Tissue Loss ≥5%	
	OR (95% CI) ^a,b^	*p*-Value	OR (95% CI) ^a,b^	*p*-Value	OR (95% CI) ^a,b^	*p*-Value
PG-SGA score at the end of RT						
0–3	Reference		Reference		Reference	
≥4	74.07 (9.34–587.3)	<0.001	72.96 (9.45–563.18)	<0.001	8.01 (3.78–16.98)	<0.001
Any PRO-CTCAE score						
0–2	Reference		Reference		Reference	
≥3	3.63(1.43–9.17)	0.007	8.81 (3.26–20.04)	<0.001	6.67(2.94–15.12)	<0.001
PRO-CTCAE, diarrhea frequency						
0–2	Reference		Reference		Reference	
≥3	4.73 (1.85–12.13)	0.001	7.65 (3.08–19.00)	<0.001	7.62 (3.23–18.00)	<0.001
PRO-CTCAE, abdominal pain severity						
0–2	Reference		Reference		Reference	
≥3	1.13 (0.33–3.85)	0.84	3.97 (1.23–12.85)	0.02	1.98 (0.69–5.67)	0.20
PRO-CTCAE, abdominal pain interference						
0–2	Reference		Reference		Reference	
≥3	5.47 (1.88–15.93)	0.002	19.47 (5.25–72.28)	<0.001	10.12 (2.72–37.59)	0.001

^a^ Estimated through logistic regression models adjusted for NLR and bowel V45. ^b^ PG-SGA and PRO-CTCAE items were analyzed separately in multivariable logistic regression models considering interaction.

## Data Availability

The data are available to the corresponding author Jie Lee upon reasonable request.

## Supplementary Materials

The following are available online at https://www.mdpi.com/article/10.3390/nu13082629/s1, Figure S1: Mean changes with 95% confidence interval bars in BMI, SMI, and TATI from baseline to 3 months after treatment completion according to PRO-CTCAE (A) diarrhea frequency, (B) abdominal pain severity, and (C) abdominal pain interference; Table S1: Univariable logistic regression analysis of factors associated with body composition changes.
