# Supplementary material for: Association of Patient-Reported Outcomes and Nutrition with Body Composition in Women with Gynecologic Cancer Undergoing Post-Operative Pelvic Radiotherapy: An Observational Study
Source: Nutrients. 2021 Jul 29;13(8):2629. doi: 10.3390/nu13082629 (PMC8399258; doi:10.3390/nu13082629)
Supplement: Supplementary file 1 [file nutrients-13-02629-s001.zip › nutrients-1299811-supplementary.pdf]

Supplementary Material

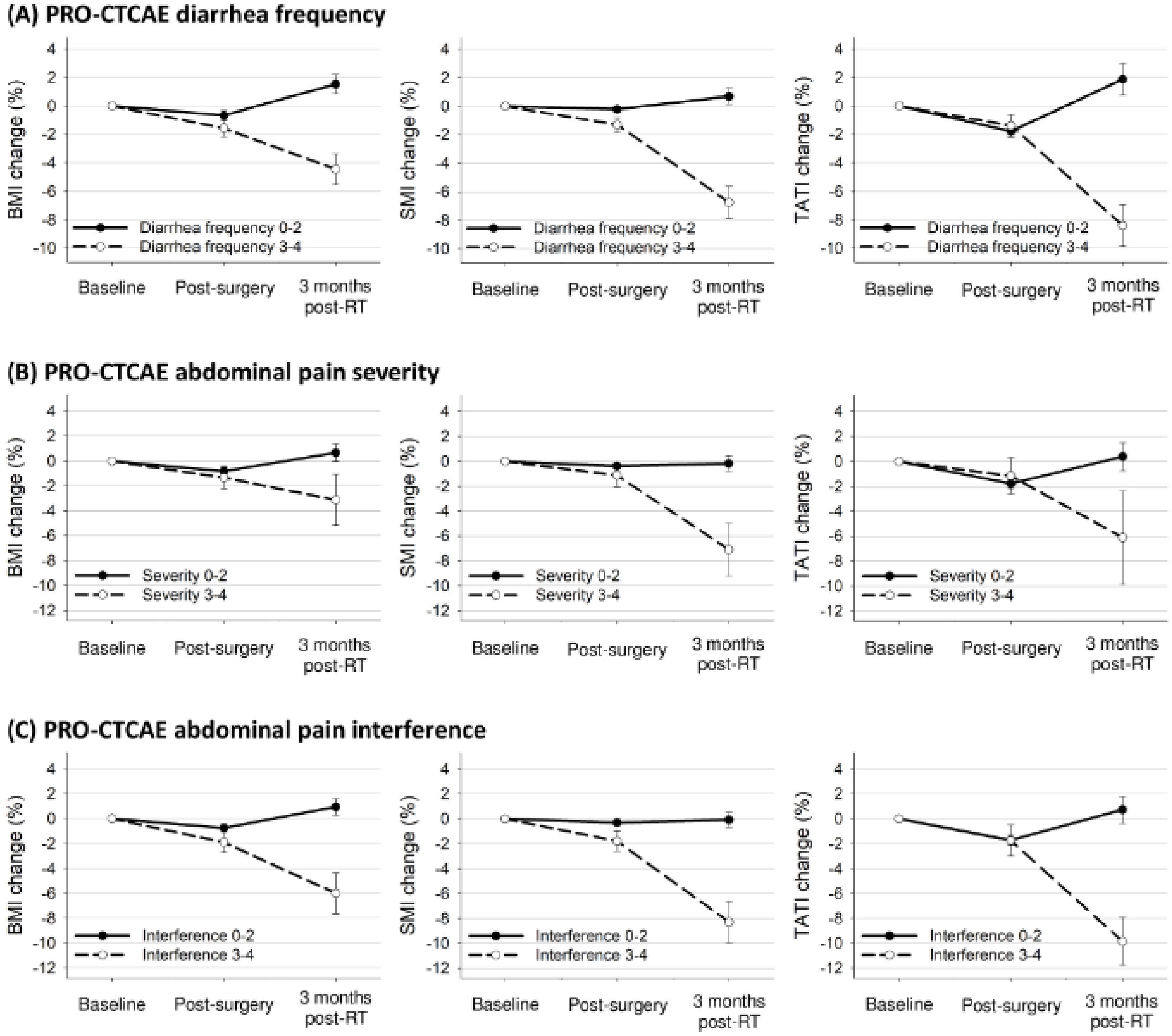

Figure S1. Mean changes with 95% confidence interval bars in BMI, SMI, and TATI from baseline to 3 months after treatment completion according to PRO-CTCAE (A) diarrhea frequency, (B) abdominal pain severity, and (C) abdominal pain interference. BMI, body mass index; PRO-CTCAE, patient-reported outcome version of the Common Terminology Criteria for Adverse Events; RT, radiotherapy; SMI, skeletal muscle index; TATI, total adipose tissue index.

Table S1 Univariable logistic regression analysis of factors associated with body composition changes.

	Weight loss ≥5%		Muscle loss ≥5%		Adipose tissue loss ≥5%	
**Variable**	**OR (95% CI)**	***p*-value**	**OR (95% CI)**	***p*-value**	**OR (95% CI)**	***p*-value**
**Age**	1.03 (0.99-1.07)	0.18	1.01 (0.97-1.04)	0.75	1.03 (0.99-1.06)	0.09
**Disease cite**						
Endometrium	Reference		Reference		Reference	
Cervix	2.08 (0.97-4.47)	0.06	1.51 (0.74-3.08)	0.25	1.58 (0.86-2.91)	0.14
**Surgery type**						
Open	Reference		Reference		Reference	
Minimally invasive	0.46 (0.15-1.39)	0.17	0.87 (0.37-2.04)	0.75	0.83 (0.40-1.70)	0.60
**Chemotherapy**						
No	Reference		Reference		Reference	
Yes	1.33 (0.60-2.92)	0.48	1.29 (0.64-2.58)	0.47	0.96 (0.53-1.75)	0.90
**NLR**						
≤3	Reference		Reference		Reference	
>3	3.63 (1.66-7.94)	0.001	3.40 (1.67-6.94)	0.001	2.58 (1.41-4.71)	0.002
**PG-SGA score at the beginning of RT**						
0-3	Reference		Reference		Reference	
≥4	0.87 (0.28-2.71)	0.81	1.77 (0.72-4.36)	0.21	1.18 (0.51-2.69)	0.70
**PG-SGA score at the end of RT**						
0-3	Reference		Reference		Reference	
≥4	91.62 (12.16-690.55)	<0.001	140.2 (18.63-1054.8)	<0.001	11.05 (5.59-21.83)	<0.001
**Any PRO-CTCAE score**						
0-2	Reference		Reference		Reference	
≥3	6.34 (2.85-14.13)	<0.001	17.50 (7.704-39.80)	<0.001	9.87 (4.74-20.53)	<0.001
**PRO-CTCAE, diarrhea frequency**						
0-2	Reference		Reference		Reference	
≥3	7.87 (3.48-17.80)	<0.001	16.71 (7.36-37.97)	<0.001	11.38 (5.21-24.87)	<0.001
**PRO-CTCAE, abdominal pain severity**						
0-2	Reference		Reference		Reference	
≥3	2.70 (0.95-7.66)	0.06	8.68 (3.26-23.13)	<0.001	3.78 (1.46-9.75)	0.006
**PRO-CTCAE, abdominal pain interference**						
0-2	Reference		Reference		Reference	
≥3	8.54 (3.13-23.30)	<0.001	24.90 (7.65-81.06)	<0.001	15.04 (4.20-53.80)	<0.001
**Physician-reported CTCAE grade**						
0-2	Reference		Reference		Reference	
≥3	1.98 (0.60-6.56)	0.26	1.46 (0.45-4.80)	0.53	0.99 (0.33-2.98)	0.98
**Bowel V45[a] (per 1 mL)**	1.015 (1.008-1.023)	<0.001	1.029 (1.018-1.039)	<0.001	1.013 (1.007-1.019)	<0.001

RT, radiotherapy
a V45 = volume (mL) of bowel bag receiving 45 Gy or more.
